# Atrial Flutter in the Elderly Patient: The Growing Role of Ablation in Treatment

**DOI:** 10.7759/cureus.50096

**Published:** 2023-12-07

**Authors:** Pawel Borkowski, Natalia Nazarenko, Shaunak Mangeshkar, Natalia Borkowska, Nikita Singh, Vibhor Garg, Matthew Parker, Ahmad Moayad Naser

**Affiliations:** 1 Internal Medicine, Albert Einstein College of Medicine, Jacobi Medical Center, New York, USA; 2 Pediatrics, Samodzielny Publiczny Zakład Opieki Zdrowotnej (SPZOZ), Krotoszyn, POL

**Keywords:** congestive cardiac faliure, cryo ablation, radiofrequency ablation (rfa), elderly population, atrial flutter

## Abstract

The prevalence of atrial flutter (AFL) is increasing among the elderly population, and managing this condition presents specific challenges within this demographic. As patients age, they often exhibit reduced responsiveness to conservative treatment, necessitating a more invasive approach. We present a case of a 93-year-old female who presented to the hospital with acute decompensated heart failure (ADHF) and AFL. A year prior, she was diagnosed with arrhythmia-induced cardiomyopathy. Despite recovering her ejection fraction (EF) through guideline-directed medical therapy (GDMT), her EF deteriorated again. The patient declined invasive management for her arrhythmia on multiple occasions. Managing such patients is challenging since the approach with pharmacotherapy alone often fails to maintain sinus rhythm or adequately control the ventricular rate. Growing evidence shows that invasive management, especially ablation, may be a safe and effective procedure for this patient population. Furthermore, the studies suggest that ablation may yield particular benefits for patients with simultaneous heart failure and atrial fibrillation/AFL (AF/AFL). Unfortunately, limited data exist regarding the invasive management of AFL in the elderly. Therefore, this case report aims to provide a comprehensive review of the current evidence regarding the safety and efficacy of ablation as a therapeutic option for AFL in elderly patients, with a particular focus on how patients with concomitant heart failure may benefit from ablation.

## Introduction

Atrial flutter (AFL) is a supraventricular arrhythmia characterized by regular atrial depolarizations (originating in a macrore-entrant circuit) at an average rate of 300 beats per minute. Typically, there is a 2:1 conduction across the atrioventricular (AV) node, leading to a regular ventricular rate of approximately 150 beats per minute. AFL is classified as typical (re-entrant loop passing through the cavotricuspid isthmus) and atypical (re-entrant loop can involve any part of the right or left atria) [1]. The incidence of AFL in the USA is estimated to be 200,000 new cases annually, and individuals aged 80 years or older experience a 100 times higher incidence of AFL than those younger than 50 years old [2]. Furthermore, the data indicates that a 65-year-old individual in the year 2050 will have a life expectancy of approximately 19.8 additional years [3]. According to the US Census Bureau, it was estimated that 0.07 million persons had AFL only, and 0.19 million had concomitant AF and AFL in 2005, but the projected prevalence for 2050 is 0.15 million for AFL only, and 0.44 million for AF and AFL [4]. This suggests that the clinical scenario of an elderly individual with AFL will become increasingly common. Hence, it is crucial to establish treatment strategies tailored to the elderly, considering their individualized care goals and prioritizing their quality of life.

Pharmacological interventions are often inadequate in maintaining sinus rhythm or achieving optimal ventricular rate control within the elderly population. Consequently, there is a growing reliance on ablation as a pivotal therapeutic approach for this demographic. Historically, the application of ablative therapy in “very elderly” patients was infrequent, primarily due to concerns regarding their overall health, capacity to tolerate the treatment, and safety of the procedure. However, ablation is gaining increasing recognition as a highly effective and safe intervention, with success rates exceeding 90%, even among elderly individuals [5]. This shift toward ablation is substantiated by a growing body of evidence highlighting its superior outcomes in comparison to pharmacotherapy, particularly among patients experiencing concurrent heart failure and AF and/or AFL.

## Case presentation

A 93-year-old African American woman with a history of non-obstructive coronary artery disease, heart failure with improved ejection fraction (EF 55%), and AFL presented to the emergency department with palpitations and shortness of breath. Her vital signs were: temperature 36.6°C, blood pressure 172/111 mmHg, pulse rate 120 bpm, and respiratory rate 18 breaths/min. Physical examination revealed bibasilar crackles, bilateral pitting edema, and warm skin. Her home medications included metoprolol 200mg once daily, sacubitril/valsartan 97mg/103mg twice daily, isosorbide dinitrate 20mg three times daily, hydralazine 25mg three times daily, and apixaban 2.5mg twice daily. She had a previous hospitalization one year ago for Acute Decompensated Heart Failure (ADHF), where she was found to have EF of 40% and new-onset AFL. After discharge, her EF improved to 55%, but she had five readmissions for ADHF.

**Figure 1:**
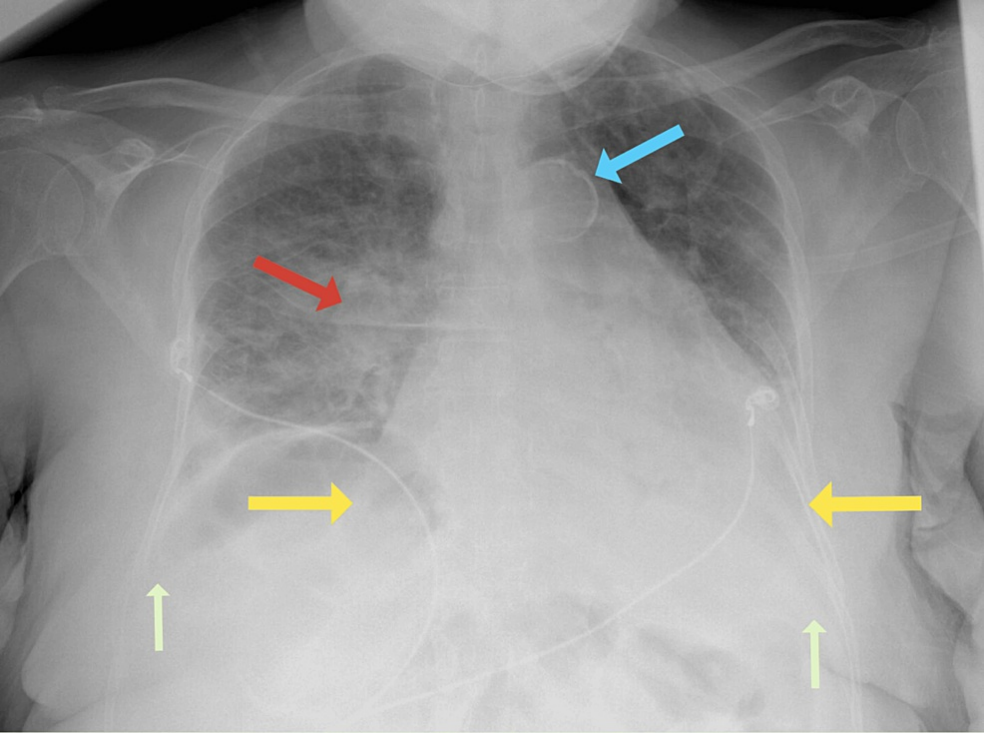
Chest radiography at the time of admission. Enlarged cardiac silhouette (yellow arrows). Calcified aorta (blue arrow). Increased central perihilar pulmonary vascular congestion (red arrow). Pleural effusions (left greater than right) are not excluded (green arrows). Decreased inspiratory effort. No pneumothorax.

Bedside ultrasound showed a moderately reduced EF, a left atrium diameter of 3.4 cm, and bilateral B-lines in the lungs. Laboratory results included elevated creatinine (1.9 mg/dL) and NT-proBNP (6,120 pg/mL). Chest x-ray revealed an enlarged cardiac silhouette, pulmonary congestion, and bilateral pleural effusions (Figure 1). EKG showed a typical AFL with a rate of 150 bpm.

After the potential precipitants of ADHF were ruled out (Table 1), the diagnosis of AFL with a rapid ventricular response was confirmed as the primary inciting factor. Aggressive diuresis improved her condition, with decreased NT-proBNP (3,164 pg/mL) and creatinine (1.3 mg/dL). She experienced persistent AFL, which was managed with metoprolol and amiodarone, but her EF declined to 35% on the official echocardiogram. Ablation was discussed again but declined by the patient. She was discharged on metoprolol succinate 200 mg once daily, sacubitril/valsartan 97mg/103mg twice daily, spironolactone 25 mg once daily, empagliflozin 10 mg once daily, isosorbide dinitrate 40 mg three times daily, hydralazine 50 mg three times daily, apixaban 2.5 mg twice daily, and amiodarone 200 mg daily. One month later, she returned with ADHF due to AFL with rapid ventricular response (NT-proBNP 22,400 pg/mL).

**Table 1:** Common precipitants of heart failure. The precipitating factor cannot be found in up to 40% of patients [11].

Common precipitants of heart failure	Occurrence (%)
Pneumonia/respiratory process [6,7,8]	28.2
Excessive salt intake [6]	22
Arrhythmias [7,9]	13-21.7
Nonsteroidal Anti-Inflammatory Drugs (NSAIDs) [9,10]	19
Improper use of antiarrhythmic or cardiodepressant agents [6,9]	15
Renal dysfunction and electrolyte disturbances [7,9]	14.7
Uncontrolled hypertension [7,9]	2-14.5
Noncompliance to medical regimen [9]	7
Myocardial ischemia [9]	2
Anemia [9]	Unknown
Thyroid disorders [9]	Unknown

## Discussion

Classically, the treatment of AFL consists of ventricular rate control, restoration of sinus rhythm, maintenance of sinus rhythm, and anticoagulation [12]. The pharmacologic management of arrhythmias in the elderly population presents challenges (Table 2). Consequently, the authors anticipate an increased dependence on invasive therapies, such as electric cardioversion and ablation, within this population. The latter is the primary focus of this article.

**Table 2:** Causes of ineffective rate and rhythm control with pharmacotherapy in the elderly population.

Age-related changes	Effect
Reduction in muscle mass and total body water, elevation in body fat [13,14]	Increased volume of distribution for lipophilic drugs [13,14]
Reduction in the surface area of intestinal epithelial cells and splanchnic blood flow, delayed gastric emptying [15,16]	Decreased absorption of medications from the gastrointestinal tract [15,16]
Reduction in liver mass and blood flow, reduced serum albumin levels [17,18]	Bioavailability of drugs subject to first-pass metabolism increases while the bioavailability of pro-drugs decreases, decline in total drug concentration and increased fraction of free drugs within the system [14,18]
Cardiac fibrosis, dysfunctional adrenoceptors and ion channels, electrical remodeling [19]	Arrhythmias [19]
Hyalinization of afferent arterioles, reduction in the number of nephrons [20]	Decrease the renal excretion of drugs [20]
Cognitive impairment, insufficient social support [21,22]	Poor compliance with therapy [21,22]
Polypharmacy, drug interactions [23,24]	Increased risk for adverse drug events and hospital readmissions, higher mortality rates [23,25,26]

limited data exist regarding the ablation of AFL in the elderly. Historically, ablative therapy was rarely applied to “very elderly” patients due to concerns about their overall health status and ability to tolerate the treatment. The prevalent form of AFL is characterized by a counterclockwise macrore-entrant circuit traversing cavotricuspid isthmus [27]. By positioning an ablation line in this region, it is possible to terminate AFL and prevent its recurrence effectively. Achieving a complete isthmus block is the most reliable indicator of long-term success [28]. Ablation utilizes an alternating current (radiofrequency) or pressurized cryo-refrigerant, usually nitrous oxide (cryoablation). Some evidence suggests that cryoablation may emerge as the dominant method in the future owing to its lower cost and shorter procedure duration [29].

The safety of ablation procedures among elderly individuals is increasingly acknowledged [30,31]. However, compared to younger populations, the procedure may carry a relatively less favorable profile of side effects (e.g., cerebrovascular events, bleeding) [32]. There is compelling evidence demonstrating the effectiveness of ablation as a therapeutic intervention. For example, in the context of AFL, a comprehensive meta-analysis encompassing 21 studies revealed a success rate of 91.7% (95% CI: 88.4% to 94.9%) for a single radiofrequency ablation procedure [5]. The success rates were even higher, at 97.0% (95% CI: 94.7% to 99.4%), for multiple procedures in treating this specific arrhythmia. In addition, the data support the effectiveness of ablation among the elderly population, with some studies demonstrating comparable success rates in patients aged seventy years or older compared to younger patients (n=445 (87.4%) vs. n=742 (90.4%), respectively) [33]. Due to its safety, low procedural risk, and effectiveness, ablation is commonly employed as the initial treatment modality for attaining a sustained return to sinus rhythm. The Loire-Ardèche-Drôme-Isère-Puy-de-Dôme (LADIP) trial demonstrated that radiofrequency ablation should be prioritized over amiodarone due to its lower incidence of side effects (n=0 (0%) vs. n=5 (10%), respectively) and higher long-term success rate (n=50 (96.2%) vs. n=36 (80.5%), respectively) [34]. Additionally, in the 2019 Catheter Ablation vs Antiarrhythmic Drug Therapy for Atrial Fibrillation (CABANA) trial, catheter ablation emerged as an economically attractive option compared to drug therapy for the treatment of AF [35].

Patients with AFL and heart failure may represent a subgroup that can experience substantial benefits from ablation. However, it is essential to acknowledge that existing data predominantly concentrates on ablation's impact on AF, leaving AFL outcomes relatively underrepresented. For instance, in a comprehensive study involving patients with heart failure and AF, Pallisgaard et al. reported a higher reduction in all-cause mortality after ablation than in medical management (4.7% (2.3%-7.2%) vs. 3.7% (1.0%-6.3%), respectively) [36]. Similarly, in the CABANA trial, which encompassed patients with heart failure and AF, ablation resulted in a remarkable 43% relative decrease in all-cause mortality (hazard ratio, 0.57 [95% CI, 0.33-0.96]) when compared to medical management [37]. Furthermore, a large-scale investigation involving patients from the Korean National Health Insurance Service database demonstrated that ablation significantly reduced the risk of hospitalization for individuals with AF and heart failure when compared to medical therapy (0.7 vs. 1.9 per 100 person‐years; HR, 0.43; 95% CI, 0.37-0.50) [38]. Additionally, Wu et al. illustrated that among patients with heart failure and AF, those in the ablation group exhibited a notably lower incidence of stroke/transient ischemic attack compared to the medical management group (n=14 (4.2%) vs. n=23 (7.2%), respectively), and they reported an enhanced quality of life [39]. The current data underscores a substantial reduction in all-cause mortality, the rates of hospitalization, and TIA/stroke among individuals experiencing concomitant heart failure and AF who have undergone ablation procedures. Based on the available data, it is highly likely that similar trends would be observed among patients with concomitant heart failure and AFL [40].

The incidence of AFL is increasing in the elderly population, and treating it presents unique complexities among this group. Key challenges related to the pharmacologic management of AFL in older people are listed in Table 1. Therefore, ablation has an increasing role in managing these patients. The ablation has advanced significantly, transforming it into an efficient therapeutic approach for achieving rhythm control. The authors believe that ablation will emerge as a fundamental therapeutic modality for managing AFL, particularly in the context of geriatric patients. Unfortunately, clinical trials have often lacked adequate representation of older adults in the past, and most of them have primarily focused on AF rather than AFL. Hence, additional investigations are warranted to explore the application of the ablation procedure in the context of AFL.

## Conclusions

We are observing a growing reliance on ablation as a definite therapeutic intervention among elderly patients with AFL. Ablation is a safe and highly effective procedure among elderly patients with a success rate comparable to younger patients. Of particular note are the substantial benefits accrued by elderly individuals with concomitant heart failure and AFL who undergo ablation, leading to significant reductions in mortality, hospital admissions, and stroke risk. Given its safety, effectiveness, and benefits, ablation will likely become a primary therapeutic choice for elderly patients with AFL.
